# Polyclonal activation of naïve T cells by urease deficient-recombinant BCG that produced protein complex composed of heat shock protein 70, CysO and major membrane protein-II

**DOI:** 10.1186/1471-2334-14-179

**Published:** 2014-04-02

**Authors:** Yumiko Tsukamoto, Yumi Maeda, Toshiki Tamura, Tetsu Mukai, Masahiko Makino

**Affiliations:** 1 Department of Mycobacteriology, Leprosy Research Center, National Institute of Infectious Diseases, 4-2-1 Aobacho, Higashimurayama, Tokyo 189-0002, Japan

**Keywords:** Tuberculosis, BCG, T cell activation

## Abstract

**Background:**

*Mycobacterium bovis* bacillus Calmette-Guérin (BCG) is known to be only partially effective in inhibiting *M. tuberculosis* (MTB) multiplication in human. A new recombinant (r) urease-deficient BCG (BCG-dHCM) that secretes protein composed of heat shock protein (HSP)70, MTB-derived CysO and major membrane protein (MMP)-II was produced for the efficient production of interferon gamma (IFN-γ) which is an essential element for mycobacteriocidal action and inhibition of neutrophil accumulation in lungs.

**Methods:**

Human monocyte-derived dendritic cells (DC) and macrophages were differentiated from human monocytes, infected with BCG and autologous T cells-stimulating activity of different constructs of BCG was assessed. C57BL/6 mice were used to test the effectiveness of BCG for the production of T cells responsive to MTB-derived antigens (Ags).

**Results:**

BCG-dHCM intracellularly secreted HSP70-CysO-MMP-II fusion protein, and activated DC by up-regulating Major Histcompatibility Complex (MHC), CD86 and CD83 molecules and enhanced various cytokines production from DC and macrophages. BCG-dHCM activated naïve T cells of both CD4 and CD8 subsets through DC, and memory type CD4^+^ T cells through macrophages in a manner dependent on MHC and CD86 molecules. These T cell activations were inhibited by the pre-treatment of Ag-presenting cells (APCs) with chloroquine. The single and primary BCG-dHCM-inoculation produced long lasting T cells responsive to *in vitro* secondarily stimulation with HSP70, CysO, MMP-II and H37Rv-derived cytosolic protein, and partially inhibited the replication of aerosol-challenged MTB.

**Conclusions:**

The results indicate that introduction of different type of immunogenic molecules into a urease-deficient rBCG is useful for providing polyclonal T cell activating ability to BCG and for production of T cells responsive to secondary stimulation.

## Background

Tuberculosis is a chronic infectious disease, induced by intracellular infection with MTB and is responsible for around 1.4 million deaths yearly worldwide [1-3], and it is estimated that one-third of the global populations is latently infected with MTB. The emergence and worldwide spread of multidrug-resistant strains of MTB mandates the development of more effective preventive and therapeutic tools [4]. Studies using T cell receptor transgenic mice specific for Ag85B-derived CD4^+^ T cells epitope which is one of the most useful animal model for understanding host defense mechanisms against MTB clearly demonstrated that the MTB-susceptible APCs including DC and macrophages need 7–10 days to initiate activating type 1 CD4^+^ T cells and CD8^+^ T cells in regional lymph nodes after aerosol MTB infection, and the stimulated T cells need 4–5 weeks to initiate inhibiting the growth of MTB in primary mice [5,6]. However, after the adequate activation of both subsets of T cells, the number of MTB residing in the lung remains stable [5,6]. These observations clearly demonstrate that type 1 T cells are essential elements in inhibiting the multiplication of MTB and also that lack of memory type T cells capable of reacting to MTB-infected APCs strongly and immediately allows MTB to multiply. In addition to the animal studies, *in vitro* studies using human APCs or T cells reveal that host defense against MTB is conducted chiefly by type 1 CD4^+^ T cells and CD8^+^ T cells [7-9]. Among them, IFN-γ produced from both subsets of T cells is considered as one of the most important element for mycobacteriocidal action [10], and cytotoxic T lymphocytes differentiated from the activated CD8^+^ T cells act chiefly at late stage of MTB infection [1,11,12]. The killing process of MTB-infected APCs is via a granule-dependent mechanism [13,14]. Although BCG has been used as a vaccine against tuberculosis widely, BCG cannot prevent the manifestation of adult lung tuberculosis [15]. The major reason why BCG is not fully functional, remains to be elucidated. BCG, as a vaccine, is not convincing in terms of T cell activation, since BCG activates naïve CD4^+^ T cells substantially, but poorly activates naïve CD8^+^ T cells [16,17]. The reason why BCG cannot activate naïve T cells fully, may be based on the lack of the ability to induce phagosomal maturation [18-20]. Therefore, improvement of T cell-stimulating ability of BCG is strongly required. Presently various new protein vaccine candidates have been selected for clinical trials [18-20]. Actually the vaccine candidates including early secretory antigenic target-6, culture filtrate protein 10, Ag85 family and polyprotein Ag designated Mtb72F and a fusion protein ID93 [9,21-26] are produced based on Ags that are recognized in infected individuals. However, fully reliable new vaccine has not been established yet.

Recently, we have produced recombinant BCG, termed BCG-DHTM which strongly activated human naïve CD4^+^ T cells and naïve CD8^+^ T cells, and, in mice, produced T cells responsive to H37Rv-derived cytosolic protein [27]. In the production of BCG-DHTM, we employed two independent strategies in order to overcome the intrinsic defect of BCG, that is an ability to block phagosome maturation to inhibit processing of Ag and presentation to type 1 T cells. One of the strategies is inactivation of *ureC* gene of BCG, which encodes urease, from BCG [19,20]. The urease produces ammonia from urea and inhibits the phagosomal acidification. The urease depletion facilitates the translocation of BCG to lysosome, and enhanced the ability of BCG to activate human naïve CD4^+^ T cells [19,20]. The other one is the intracellular secretion of antigenic molecule. As the key antigen, we used MMP-II, since MMP-II is recognized by human T cells after infection with *M. leprae* or BCG, and can ligate Toll like receptor (TLR)2 and consequently activate both DC and macrophages [28-31]. Also we used HSP70, since HSP70 has a chaperon activity and can prime cytotoxic T lymphocytes. The intraphagosomal secretion of HSP70-MMP-II fusion protein induced strong activation of naïve CD4^+^ T cells and CD8^+^ T cells [32]. Since both strategies, that is urease depletion and an intracellular secretion of HSP70-MMP-II fusion protein, worked synergistically in terms of T cell activation, the gene encoding the HSP70-MMP-II fusion protein was introduced into urease-depleted rBCG (BCG-ΔUT-11-3) for production of BCG-DHTM [27].

Although BCG-DHTM activated both subsets of T cells to produce IFN-γ, production of memory T cells capable of responding to MTB-derived molecules which can be induced chiefly in the activating phase of MTB growth, is needed for the tuberculosis protection. To address this point, we selected CysO (Rv1335 or CFP10A) as the target gene, since CysO also participates in cysteine biosynthesis pathway in MTB [33]. MTB has two independent cysteine biosynthesis pathways, namely the conventional pathway and the alternative pathway. CysO is engaged in the alternative pathway. This pathway is more advantageous under the oxidative conditions. Indeed, CysO expression is induced under diamide stress, one of the oxidative stress conditions [34]. This implies that CysO may be essential for MTB to survive within macrophages, since MTB is exposed to oxidative stress in macrophages [35]. Furthermore, CysO expression is repressed in hypoxic condition and induced in reaeration condition, indicating that CysO may be essential for MTB in the growth phase [36]. Moreover CysO is categorized under ubiquitin superfamily, and may direct protein towards proteasome degradation pathway, which is essential for many cellular processes [37]. Therefore, the CysO involvement in improved cellular response against MTB within macrophages or in growth phase was considered.

In this study, we introduced CysO gene in combination with HSP70-MMP-II fusion gene into urease-deficient BCG-ΔUT-11-3, and produced new rBCG termed BCG-dHCM, and evaluated its T-cell stimulating activities.

## Methods

### Preparation of cells and Ags

Peripheral blood was obtained from healthy PPD-positive individuals under informed consent. The study was approved by the ethics committee of the National Institute of Infectious Diseases, Tokyo. In Japan, BCG vaccination is compulsory for children (0 ~ 1 year-old). Peripheral blood mononuclear cells (PBMCs) were isolated using Ficoll-Paque Plus (GE Healthcare, Uppsala, Sweden) and cryopreserved in liquid nitrogen until use, as previously described [38]. The viability of T cells obtained from cryopreserved PBMCs was more than 90% and no selection in terms of functionality was induced in both monocytes, a precursor of DC and macrophages, and T cells. For the preparation of peripheral monocytes, CD3^+^ T cells were removed from either freshly isolated heparinized blood, or cryopreserved PBMCs using immunomagnetic beads coated with anti-CD3 monoclonal antibody (mAb) (Dynabeads 450; Dynal Biotech, Oslo, Norway). The CD3^-^ PBMC fraction was plated on tissue culture plates and the non-plastic adherent cells were removed by extensive washing. The remaining adherent cells were used as monocytes [39]. Monocyte-derived DC were differentiated as described previously [38,40]. Briefly, monocytes were cultured in the presence of 50 ng of rGM-CSF (Pepro Tech EC LTD, London, England) and 10 ng of rIL-4 (Pepro Tech) per ml [40]. On day 4 of culture, immature DC were infected with rBCG at an indicated multiplicity of infection (MOI) and, on day 6 of culture, DC were used for further analyses of surface Ag and for mixed lymphocyte assays. Macrophages were differentiated as described previously [41,42]. In brief, monocytes were cultured in the presence of 10 ng of rM-CSF (R & D Systems, Inc., Minneapolis, MN) per ml. On day 5 of culture, macrophages were infected with rBCG at an indicated MOI and, on day 7 of culture, they were used for further analyses of surface Ag and mixed lymphocyte assay. The rMMP-II protein was produced as described previously [28,43], and the CysO protein was produced in an LPS-free condition by using *M. smegmatis*. Polyclonal Ab against CysO was produced by immunizing rabbit with the recombinant protein. The rHSP70 protein was purchased (Hy Test Ltd., Turku, Finland) and H37Rv-derived cytosolic protein was produced as described previously [43].

### Vector construction and preparation of rBCG

The genomic DNAs were obtained from BCG substrain Tokyo and from MTB H37Rv strain. The oligonucleotide primers used for the amplication of *hsp70* gene were F-Mb70Bal (5′-aaaTGGCCAtggctcgtgcggtcggg-3′) and R-Mb70Eco (5′-aaaGAATTCcttggcctcccggccg-3′). MMP-II sequence from MTB genomic DNA was amplified with primers: F-MMP TB Eco (aattGAATTCatgcaaggtgatcccgatgt) and R-MMP TB Sal (5′-aattGTCGACtcaggtcggtgggcgaga). CysO sequence from MTB genomic DNA was amplified with primers: F-CysO (5′-ggccgggaggccaagaacgtcaccgtatccattcc-3′) and R-CysO: (5′-atcgggatcaccttgcccaccggccacggcgggga-3′). The amplified HSP70 and MMP-II sequence were digested with appropriate restriction enzymes and cloned into parental pMV261H plasmid. For the cloning of CysO sequence into pMV261, In-Fusion HD cloning kit (Clontech laboratories, Mountain View, CA) was used. For replacing kanamycin resistance gene to hygromycin resistance cassette, the *Xba* I-*Nhe* I fragment from pYUB854 [44] was cloned into *Spe* I-*Nhe* I fragment of the plasmid [44]. The rBCG (BCG-ΔUT-11) of which *ureC* gene was disrupted, was produced as described previously [20]. The hygromycin cassette in the BCG-ΔUT-11 was removed by using pYUB870 encoding γδ-resolvase (γδ-*tnp*R) [44]. The unmarked BCG was named BCG-ΔUT-11-3. The HSP70-CysO-MMP II fusion protein expressing vector was introduced into BCG-ΔUT-11-3 by electroporation method. BCG-ΔUT-11-3 containing pMV-HSP70-CysO-MMP-II as an extrachromosomal plasmid is referred to as BCG-dHCM, and that containing pMV-261-hygromycin is referred to as BCG-261H (BCG vector control). Recombinant BCGs and MTB H37Rv strain were grown to log phase, and stored at 10^8^ colony forming unit (CFU)/ml at -80°C. Before infection to DC and macrophages, BCGs were counted by colony assay method. There is no significant difference in the *in vitro* culture growth between BCG-261H and BCG-dHCM.

### Analysis of cell surface Ag

The expression of cell surface Ag on DC was analyzed using FACSCalibur (BD Bioscience, San Jose, CA). Dead cells were eliminated from the analysis by staining with propidium iodide (Sigma-Aldrich, St. Louis, MO) and 1 × 10^4^ live cells were analyzed. For the analysis of the cell surface Ag, the following mAbs were used: FITC-conjugated mAb against HLA-ABC (G46-2.6, BD Bioscience), HLA-DR (L243, BD Bioscience), CD86 (FUN-1, BD Bioscience), and CD83 (HB15a, Immunotech, Marseille, France).

### APC function of DC

The ability of DC infected with BCG or pulsed with recombinant protein and BCG-infected macrophages to stimulate T cells was assessed using an autologous APC-T cell co-culture as previously described [40,45]. Purification of CD4^+^ and CD8^+^ T cells was conducted by using negative-isolation kits (Dynabeads 450, Dynal Biotech) [40]. The purity of the CD4^+^ and CD8^+^ T cells was more than 95% when assessed using FACSCalibur. Naïve CD4^+^ and CD8^+^ T cells were produced by further treatment of these T cells with mAb to CD45RO, which were followed by beads coated with goat anti-mouse IgGs Ab (Dynal Biotech). The purity of both subsets of naïve T cells was more than 97%. However, there was no contamination of memory type T cells in the naïve T cell preparations. More than 98% of CD45RA^+^ T cells was positive in the expression of CCR7 molecule. Memory type T cells were similarly produced by the treatment of cells with mAb to CD45RA Ag. The purified responder cells (1 × 10^5^ per well) were plated in 96-well round-bottom tissue culture plates, and APCs infected with rBCG or pulsed with protein were added to give the indicated APC: T cell ratio. Supernatants of APC-T cell co-cultures were collected on day 4 and the cytokine levels were determined. In some cases, rBCG-infected DC and macrophages were treated with mAb to HLA-ABC (W6/32, Mouse IgG2a, kappa), HLA-DR (L243, Mouse IgG2a, kappa), CD86 (IT2.2, Mouse IgG2b, kappa, BD Biosciences) or normal mouse IgG. Also, in some cases, immature DC and macrophages were treated with 50 μM of chloroquine (Sigma-Aldrich) for 2 h and subsequently infected with BCG-dHCM. The optimal dose of the Abs and reagents was determined in advance.

### Measurement of cytokine production

Levels of the following cytokines were measured; IFN-γ produced by CD4^+^ and CD8^+^ T cells, and IL-12p70, IL-12p40, TNFα, IL-1β and GM-CSF produced by DC or macrophages stimulated for 24 or 48 h with rBCGs. The concentrations of these cytokines were quantified using the enzyme assay kits, Opt EIA Human ELISA Set (BD Bioscience).

### Animal studies

For inoculation into mice, rBCG and MTB H37Rv strain were cultured in Middlebrook 7H9 medium supplemented with Middlebrook ADC enrichment to log phase and stored at 10^8^ CFU/ml at -80°C. Before the aliquots were used for inoculation, the concentration of viable bacilli was determined by plating on Middlebrook 7H10 agar plate supplemented with Middlebrook OADC enrichment. Three 5-week-old C57BL/6 J mice (Clea Japan Inc., Tokyo, Japan) per group were inoculated subcutaneously with 0.1 ml of PBS or PBS containing 1 × 10^3^ rBCGs. The animals were kept in specific pathogen free conditions and were supplied with sterilized food and water. Four or 12 weeks after inoculation, the spleens were removed and the splenocytes were suspended at a concentration of 2 × 10^6^ cells per ml in culture medium. The splenocytes were stimulated with an indicated concentration of rMMP-II, rHSP70 (HyTest), rCysO or H37Rv-derived cytosolic protein in triplicates in 96-well round bottom microplates [20,30]. The individual culture supernatants were collected 3–4 days after stimulation and IFN-γ was measured using Opt EIA Mouse ELISA Set (BD Bioscience). For observing the effect of BCG vaccination on MTB infection, five C57BL/6 mice per group were vaccinated with 1 × 10^3^ CFU/mouse either BCG-261H or BCG-dHCM for 6 weeks, and were challenged with 100 CFU/lungs of H37Rv by aerosol infection using an automated inhalation exposure apparatus (Glas-Col Corp., IN, Model 099C A4212). Six weeks later, bacterial burden in lungs and spleen was assessed by mechanical disruption in PBS with 0.05% v/v Tween 80 and enumerated by colony assay. Animal studies were reviewed and approved by the Animal Research Committee of Experimental Animals of the National Institute of Infectious Diseases, and were conducted according to their guidelines.

### Statistical analysis

Student’s *t*-test was applied to determine the statistical differences.

## Results

### Immunological characterization of rCysO protein

**Figure 1:**
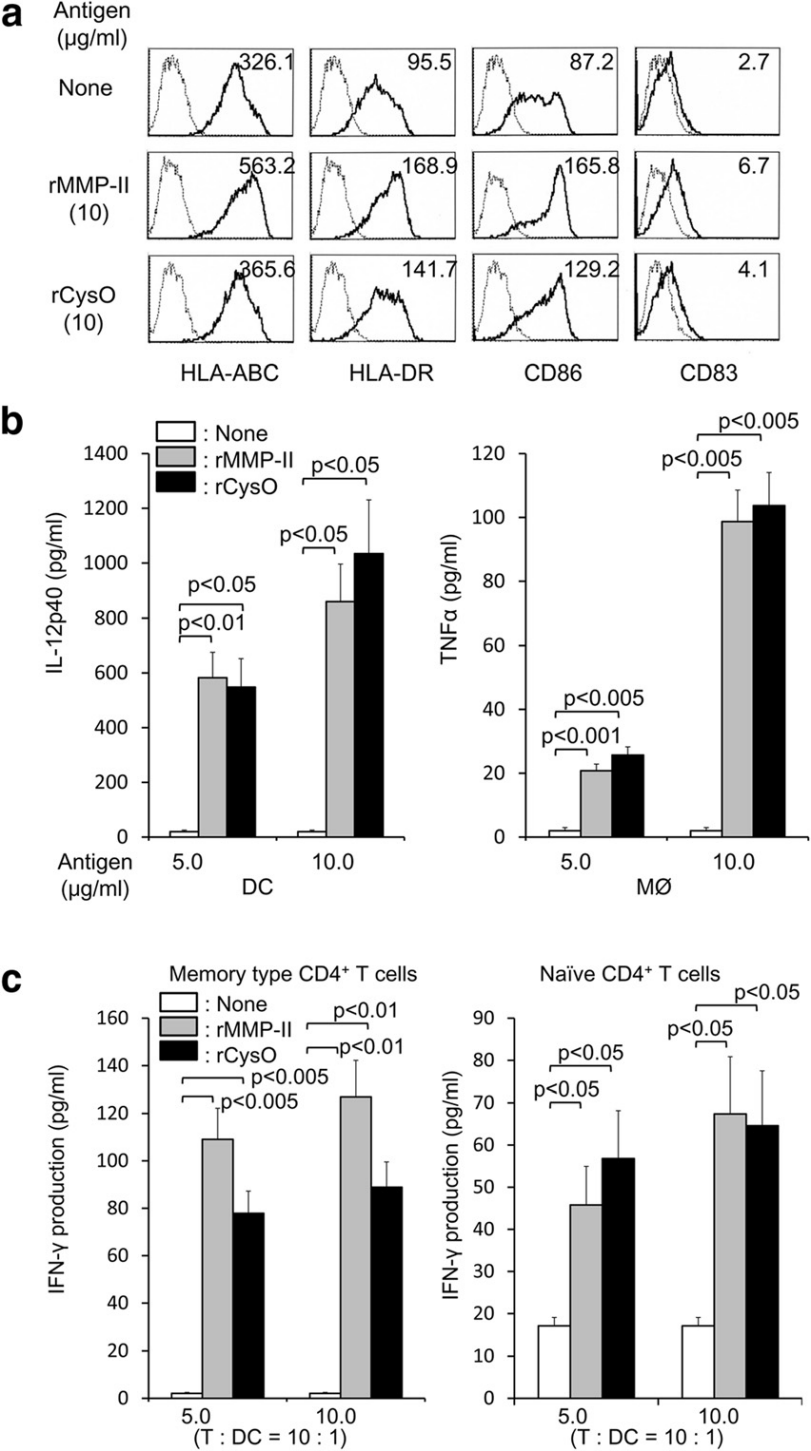
**Activation of APCs by rCysO in terms of phenotypic changes and cytokine production. a.** Up-regulated expression of APC-associated molecules on DC by stimulation with rCysO protein. Monocyte-derived immature DC were stimulated with either rMMP-II or rCysO protein at 10 μg/ml, and cultured for another 2 days in the presence of rGM-CSF and rIL-4. The DC from day 6 were gated and analyzed. Dotted lines, isotype-matched control IgG; solid lines, the indicated test mAb. Representative results of three separate experiments are shown. The number at the top right-hand corner of each panel represents the difference in mean fluorescence intensity between the control IgG and the test mAb. **b.** Cytokine production from APCs by stimulation with rCysO protein. DC produced using rGM-CSF and rIL-4 and macrophages differentiated from monocytes by using M-CSF were stimulated with either rMMP-II or rCysO protein for 24 h. **c.** IFN-γ production from CD4^+^ T cells by stimulation with rCysO protein. Monocyte-derived DC were stimulated with either rMMP-II or rCysO protein, and were used as a stimulator of memory type or naïve CD4^+^ T cells in a 4-day culture. 10^5^ responder T cells were stimulated with the protein-pulsed DC at T: DC = 10: 1. In case of the stimulation of naïve CD4^+^ T cells, the protein-pulsed DC were further treated with 1.0 μg/ml of rCD40 ligand for 24 h. The concentration of the indicated cytokine was determined by the ELISA method. A representative of three separate experiments conducted by using 3 different PBMCs-donors is shown. Assays were performed in triplicate and the results are expressed as the mean ± SD. Titers were statistically compared using Student’s *t*-test.

Since rCysO protein produced in *M. smegmatis* revealed a single band on SDS-PAGE electrophoresis and confirmed by Western blot analysis using polyclonal Ab to CysO (not shown), the immunostimulatory activities of rCysO protein were assessed using rMMP-II of MTB as a positive control. We assessed the activation of APCs from the aspect of phenotypic changes and cytokine production (Figure 1). The stimulation of immature DC with rCysO protein up-regulated the expression of MHC molecules, CD86, and CD83 Ags, comparable to rMMP-II protein (Figure 1a). Further, both rMMP-II and rCysO proteins induced IL-12p40 production from DC and TNFα production from macrophages to a similar level (Figure 1b). These results indicate that CysO protein has an ability to activate APCs. When we pulsed immature DC with rMMP-II or rCysO protein and used as a stimulator of autologous CD4^+^ T cells, both proteins induced IFN-γ production from not only memory type CD4^+^ T cells, but also from naïve CD4^+^ T cells with the help of CD40-CD40L interaction. The concentration of IFN-γ released from CD4^+^ T cells by the stimulation with rMMP-II and rCysO was comparable (Figure 1c). Therefore, rCysO protein was found to possess immunostimulatory. We also examined if the newly produced recombinant BCG-dHCM secretes the HSP70-CysO-MMP-II fusion protein by using Western blot analyses. When probed by the Ab to either of HSP70, CysO or MMP-II, BCG-dHCM showed distinct band at 95 kD equivalent to the molecular mass of the fusion protein comprising HSP70, CysO and MMP-II (not shown).

### Activation of Ag-presenting cells by BCG-dHCM

**Figure 2:**
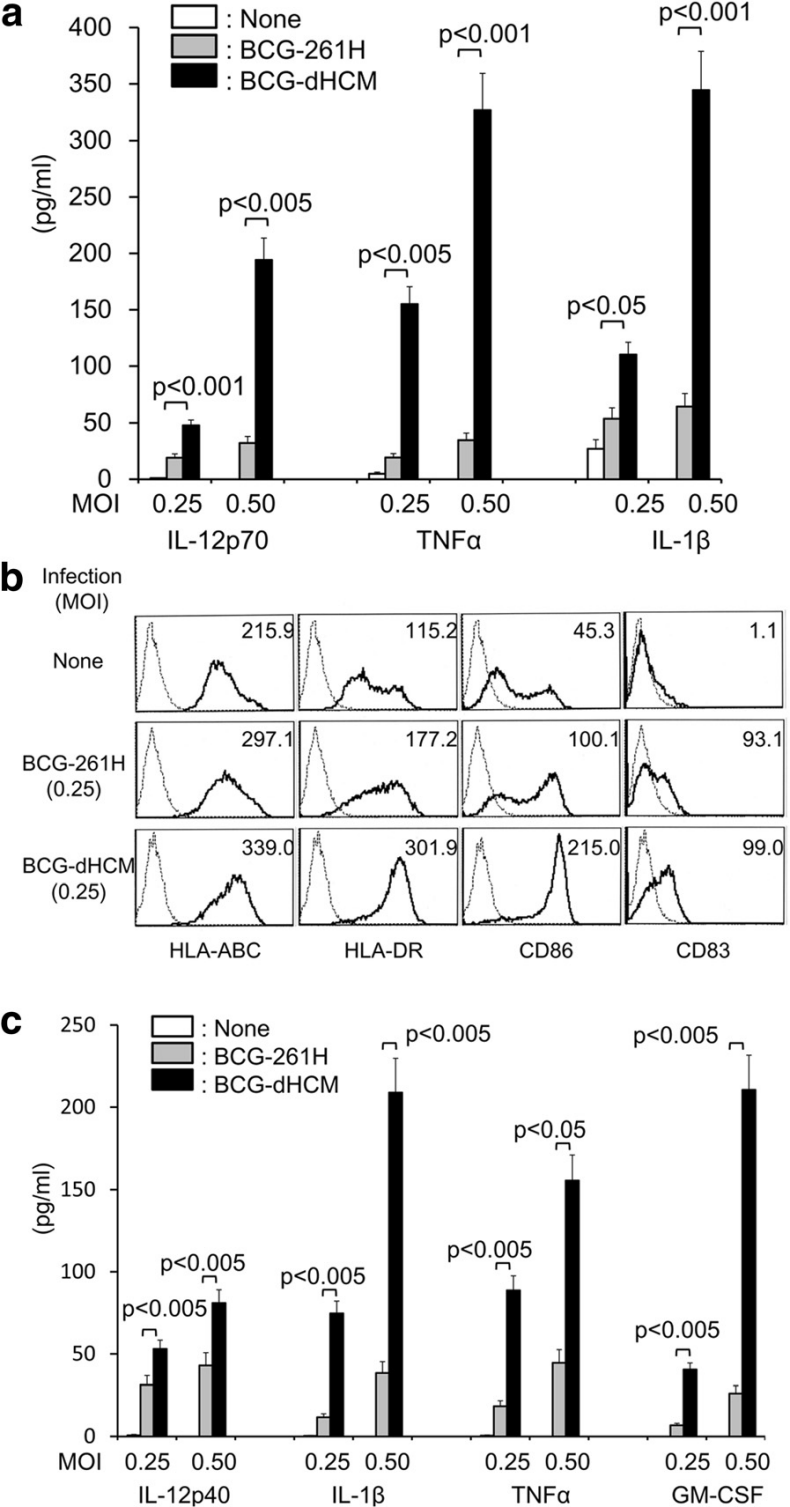
**Activation of APCs by BCG-dHCM. a.** Cytokine production from DC by stimulation with BCG-dHCM. DC produced using rGM-CSF and rIL-4 were stimulated with either BCG-261H or BCG-dHCM for 24 h. **b.** Up-regulation of APC-associated molecules and activation marker on DC by infection with BCG-dHCM. Monocyte-derived immature DC were infected with either BCG-261H or BCG-dHCM at an MOI 0.25 and cultured for another two days in the presence of rGM-CSF and rIL-4. The DC from day 6 of culture were gated and analyzed. Dotted lines, isotype-matched control IgG; solid lines, the indicated test mAb. The number at the top right corner of each panel represents the difference in the fluorescence intensity between the control IgG and the test mAb. **c.** Cytokine production from macrophages by stimulation with BCG-dHCM. Macrophages differentiated from monocytes by using M-CSF were stimulated with either BCG-261H or BCG-dHCM for 24 h. The concentration of the indicated cytokine was determined by the ELISA method. A representative of three separate experiments conducted by using 3 different PBMCs-donors is shown. Assays were performed in triplicate and the results are expressed as the mean ± SD. Titers were statistically compared using Student’s *t*-test.

In order to activate naïve T cells and produce memory type T cells efficiently, rBCG should also activate APC adequately. We assessed the activation of DC from the aspects of cytokine production and phenotypic changes (Figure 2). BCG-dHCM stimulated DC to produce IL-12p70, TNFα and IL-1β more efficiently than BCG-261H at both MOIs: 0.25 and 0.50 (Figure 2a). To assess the phenotypic changes induced by BCG-dHCM infection, we assessed the expression of MHC, CD86 and CD83 molecules on DC (Figure 2b). Both BCG-261H and BCG-dHCM up-regulated the expression of these molecules, but the infection with BCG-dHCM induced higher level of the expression. We used various dose of rBCGs for the assessment, and the similar changes were observed (not shown). Further, the stimulation of macrophages with BCG-dHCM induced the production of higher level of IL-12p40, IL-1β, TNFα and GM-CSF than that with BCG-261H at both MOIs: 0.25 and 0.50 (Figure 2c).

### Activation of naïve and memory type T cells by BCG-dHCM

**Figure 3:**
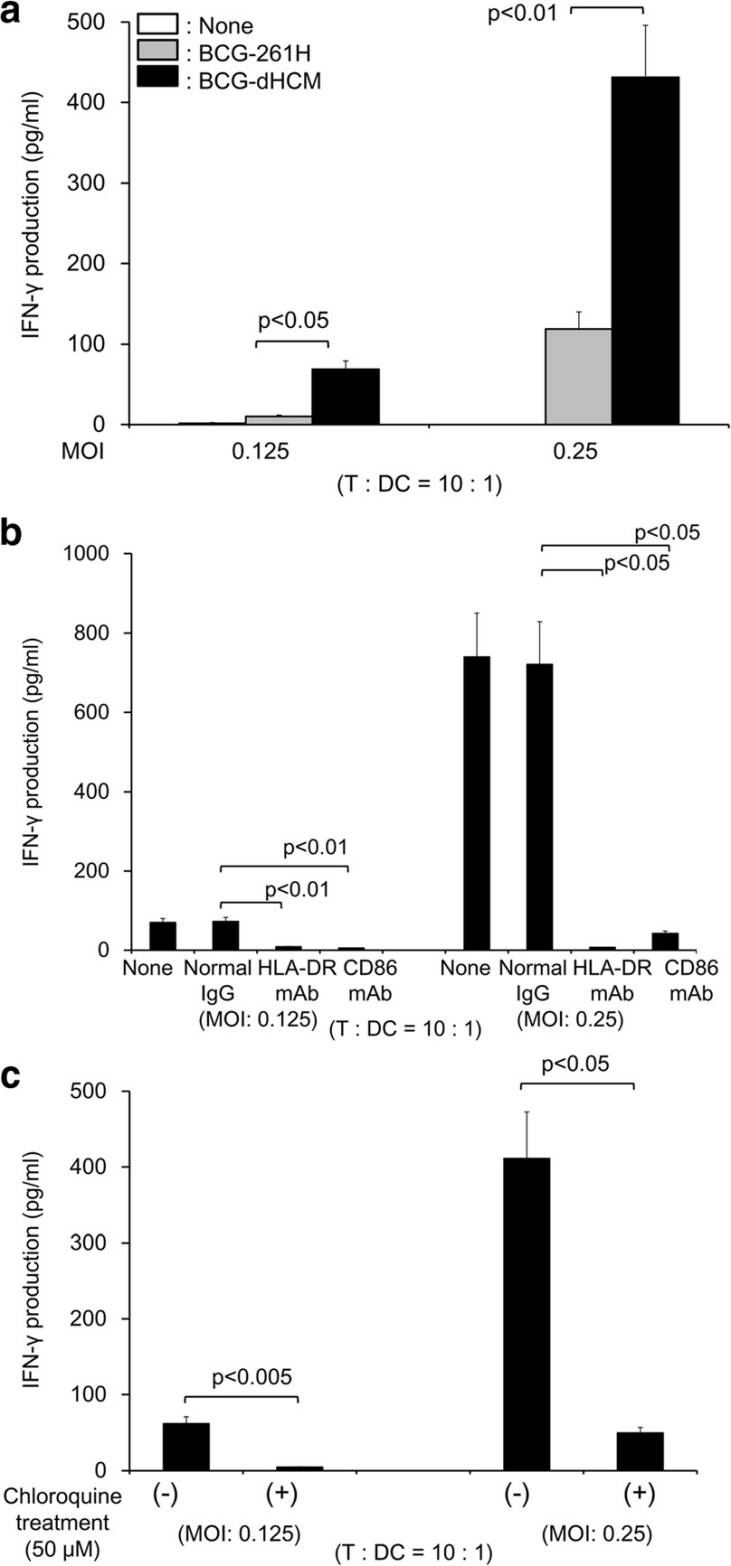
**Activation of naïve CD4**^**+ **^**T cells by BCG-dHCM through activation of DC. a.** IFN-γ production from naïve CD4^+^ T cells by stimulation with BCG-dHCM. Monocyte-derived DC were infected with either BCG-261H or BCG-dHCM at the indicated MOI, and were used as a stimulator. 10^5^ responder naïve CD4^+^ T cells were stimulated with the rBCG-infected DC at T: DC = 10: 1. **b.** Inhibition of T cell activation by the treatment of BCG-dHCM-infected DC with mAb. DC were infected with BCG-dHCM at the indicated MOI, and subsequently treated with 10 μg/ml of the mAb or normal murine IgG. These APCs were used as the stimulator of the indicated responder T cells (1 × 10^5^/well) at the indicated T/DC ratios for 4 days. IFN-γ produced by T cells was measured. **c.** Effect of chloroquine treatment of DC on the activation of T cells. Immature DC were treated with chloroquine (50 μM, 2 h) or untreated, and subsequently infected with BCG-dHCM at the indicated MOI. These DC were used as the stimulator of the indicated responder T cells at the indicated responder/stimulator ratios. IFN-γ produced by T cells was measured. A representative of three separate experiments conducted by using 3 different PBMCs-donors is shown. Assays were performed in triplicate and the results are expressed as the mean ± SD. Titers were statistically compared using Student’s *t*-test.

**Figure 4:**
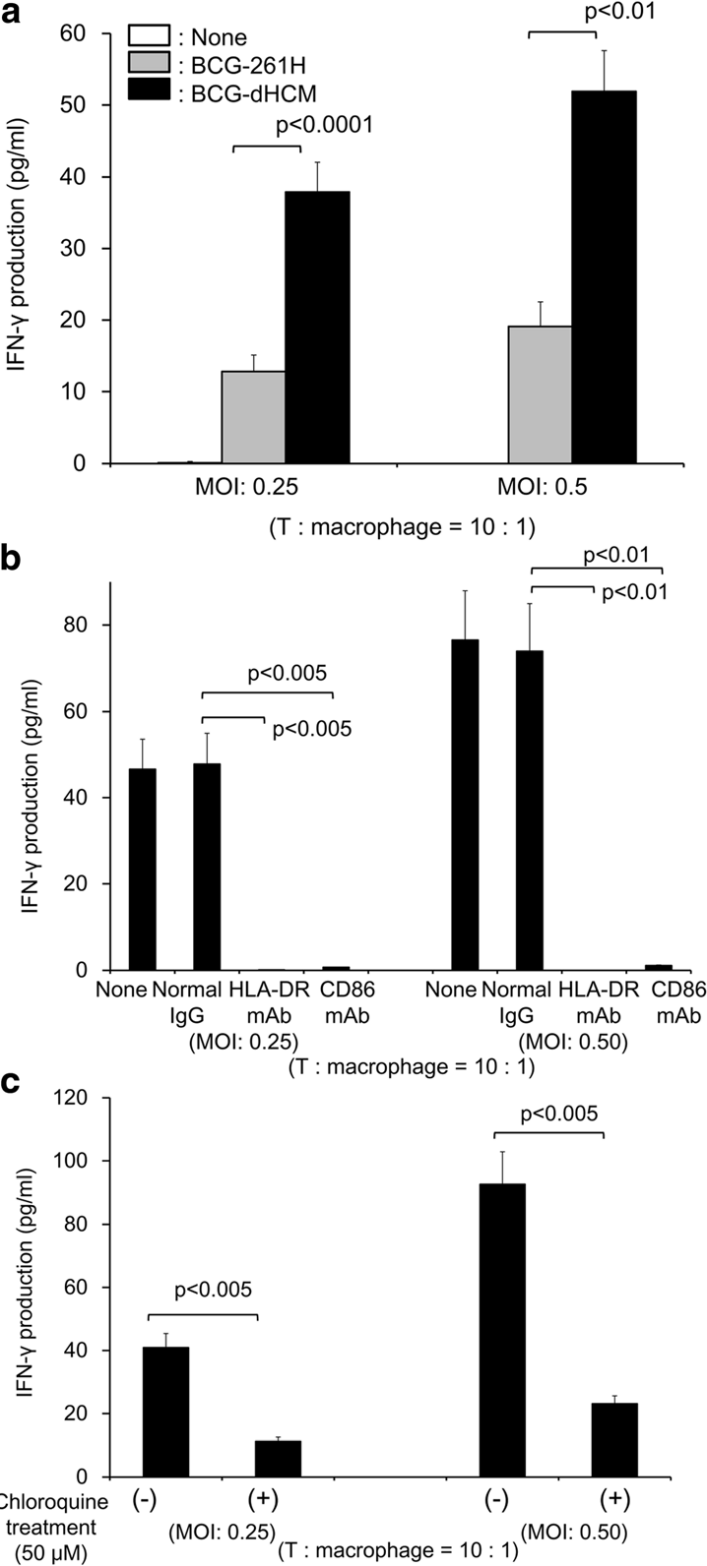
**Activation of memory-type CD4**^**+ **^**T cells by BCG-dHCM through activation of macrophages. a.** IFN-γ production from memory type CD4^+^ T cells by stimulation with BCG-dHCM. Macrophages differentiated by using M-CSF were infected with either BCG-261H or BCG-dHCM at the indicated MOI, and were used as a stimulator of responder CD4^+^ T cells in a 4-day culture. 10^5^ responder T cells were stimulated with the rBCG-infected macrophages at T: macrophage = 10: 1. **b.** Inhibition of T cell activation by the treatment of BCG-dHCM-infected macrophages with mAb. Macrophages were infected with BCG-dHCM at the indicated MOI, and subsequently treated with 10 μg/ml of the mAb or normal murine IgG. These APCs were used as the stimulator of the indicated responder T cells (1 × 10^5^/well) at the indicated T/macrophages ratios for 4 days. IFN-γ produced by T cells was measured. **c.** Effect of chloroquine treatment of macrophages on the activation of T cells. Macrophages were treated with chloroquine (50 μM, 2 h) or untreated, and subsequently infected with BCG-dHCM at the indicated MOI. These macrophages were used as the stimulator of the indicated responder T cells at the indicated responder/stimulator ratios. IFN-γ produced by T cells was measured. A representative of three separate experiments conducted by using 3 different PBMCs-donors is shown. Assays were performed in triplicate and the results are expressed as the mean ± SD. Titers were statistically compared using Student’s *t*-test.

**Figure 5:**
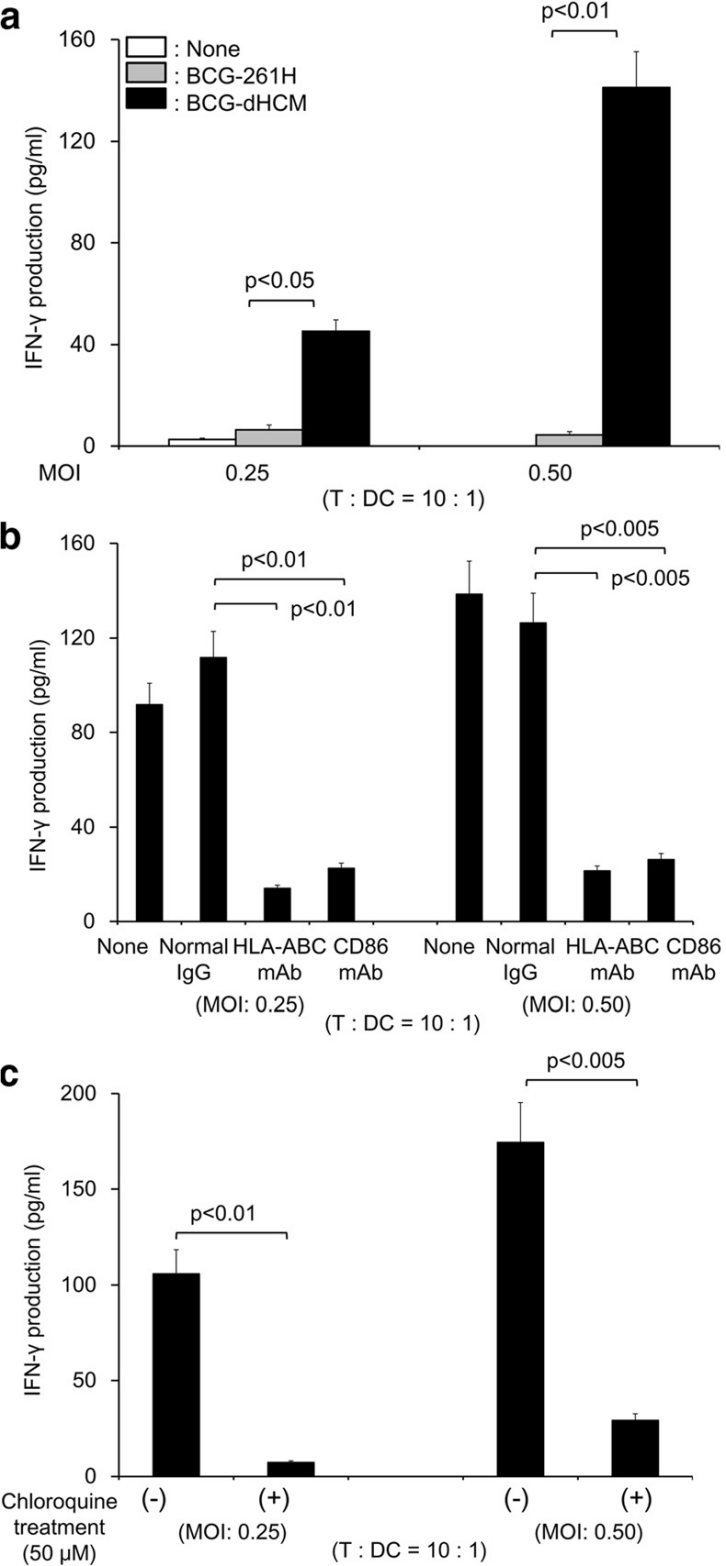
**Activation of naive CD8**^**+ **^**T cells by BCG-dHCM through activation of DC. a.** IFN-γ production from naïve CD8^+^ T cells by stimulation with BCG-dHCM. DC were infected with either BCG-261H or BCG-dHCM at the indicated MOI, and were used as a stimulator. 10^5^ responder T cells were stimulated for 4 days with rBCG-infected DC at T: DC = 10: 1. **b.** Inhibition of T cell activation by the treatment of BCG-dHCM-infected DC with mAb. DC were infected with BCG-dHCM at the indicated MOI, and subsequently treated with 10 μg/ml of the mAb or normal murine IgG. These APCs were used as the stimulator of the indicated responder T cells (1 × 10^5^/well) at the indicated T/DC ratios for 4 days. IFN-γ produced by T cells was measured. **c.** Effect of chloroquine treatment of DC on the activation of T cells. Immature DC were treated with chloroquine (50 μM, 2 h) or untreated, and subsequently infected with BCG-dHCM at the indicated MOI. These DC were used as the stimulator of the indicated responder T cells at the indicated responder/stimulator ratios. IFN-γ produced by T cells was measured. A representative of three separate experiments conducted by using 3 different PBMCs-donors is shown. Assays were performed in triplicate and the results are expressed as the mean ± SD. Titers were statistically compared using Student’s *t*-test.

The ability of BCG-dHCM to activate naïve CD4^+^ T cells through DC was evaluated using BCG-261H as a control (Figure 3). BCG-dHCM more efficiently activated naïve CD4^+^ T cells than BCG-261H to produce IFN-γ (Figure 3a). The IFN-γ production from naïve CD4^+^ T cells by BCG-dHCM stimulation seems to be induced by an Ag-specific manner, since the treatment of the surface of BCG-dHCM-infected DC with mAb to HLA-DR or CD86 showed 90% inhibition (Figure 3b). Further, pretreatment of immature DC with chloroquine prior to infection with BCG-dHCM, inhibited the production of IFN-γ from naïve CD4^+^ T cells (Figure 3c). These results suggest that the phagosomal maturation is induced by BCG-dHCM infection and is closely associated with the activation of naïve CD4^+^ T cells. Previously, we reported that only when the urease-deficient rBCG, but not normal BCG, was used as a host BCG and introduced with genes encoding antigenic molecules, the rBCG could activate memory type CD4^+^ T cells through M-CSF-dependent macrophages [27]. We checked whether BCG-dHCM activates memory type CD4^+^ T cells, and found that BCG-dHCM induced the significant production of IFN-γ from the responder population (Figure 4a). Again, the activation of memory type CD4^+^ T cells by BCG-dHCM-infected macrophages seems to be dependent on the expression of MHC class II and CD86 Ags, since the treatment of rBCG-infected macrophages with mAb to HLA-DR or CD86 inhibited the IFN-γ production significantly (Figure 4b). Further, the treatment of macrophages with chloroquine prior to BCG-dHCM infection, inhibited IFN-γ production by memory type CD4^+^ T cells (Figure 4c). Next, we assessed whether BCG-dHCM could activate naïve CD8^+^ T cells when DC were enrolled as APC (Figure 5). While vector control BCG did not induce the activation of naïve CD8^+^ T cells as reported [19,20], BCG-dHCM induced significant level of activation of naïve CD8^+^ T cells (Figure 5a). A significant concentration of IFN-γ can be released from naïve CD8^+^ T cells. Also the mAb treatment of BCG-dHCM-infected DC with anti-HLA-ABC Ab or anti-CD86 Ab significantly inhibited the IFN-γ production from naïve CD8^+^ T cells (Figure 5b), and pre-treatment of immature DC with chloroquine, again, inhibited the cytokine production from the responder (Figure 5c). These results suggest that BCG-dHCM can activate not only naïve CD4^+^ T cells, but also naïve CD8^+^ T cells through DC, and also activate memory CD4^+^ T cells through macrophages in an Ag-dependent manner. Further, the phagosomal maturation is closely associated with the T cell activation.

### Production of T cells responsive to the secondary stimulation by BCG-dHCM *in vivo*

**Figure 6:**
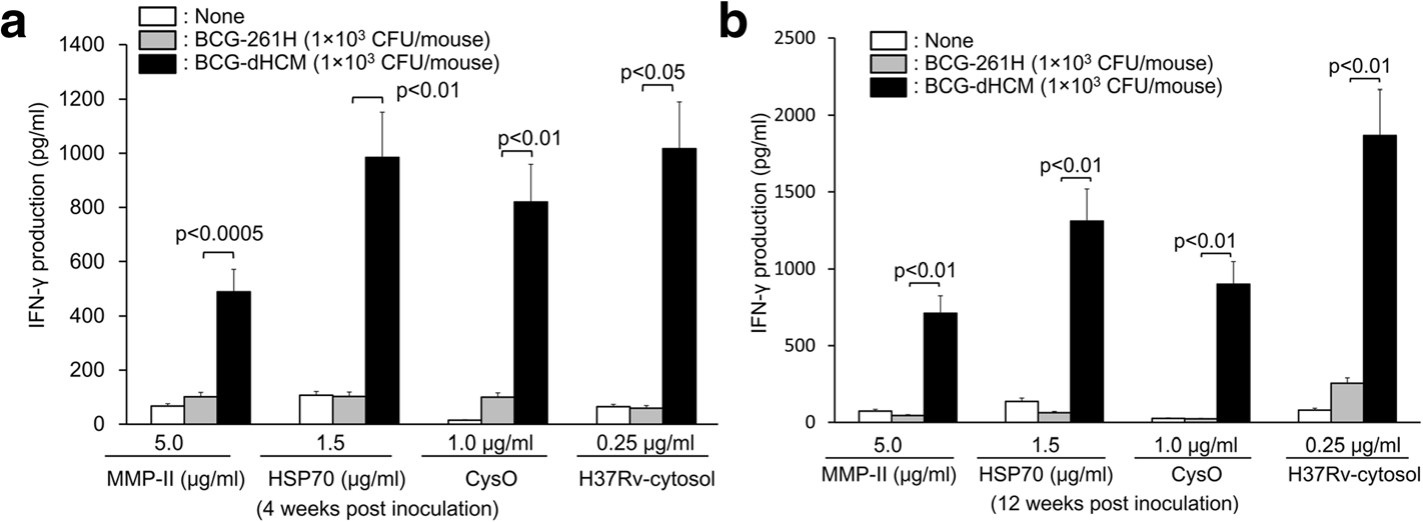
**Production of T cells responsive to the secondary stimulation in mice by infection with BCG-dHCM.** Three 5-week-old C57BL/6 mice per group were each infected with 1 × 10^3^ CFU of either BCG-261H or BCG-dHCM subcutaneously. Four **(a)** or 12 **(b)** weeks after the inoculation, splenocytes (2 × 10^5^ cells/well) were stimulated with the indicated stimulators for 4 days *in vitro*, and IFN-γ produced in the cell supernatant was measured. Assays were performed in triplicate for each mouse, and the results of three mice per group are shown as the mean ± SD. Representative results of three separate experiments are shown. Titers were statistically compared using Student’s *t*-test.

The ability of BCG-dHCM to produce T cells highly responsive to the secondary *in vitro* stimulation was examined by *in vivo* functional studies (Figure 6). C57BL/6 mice were subcutaneously inoculated with 1 × 10^3^ CFU/mouse of either BCG-261H or BCG-dHCM 4 weeks before stimulation *in vitro* (Figure 6a). Not only MMP-II, HSP70 and CysO of which encoding genes were introduced into BCG-dHCM, but also H37Rv-derived cytosolic protein were used as a secondary *in vitro* stimulator. The T cells from mice inoculated with BCG-dHCM respond more vigorously to all stimulators and produced higher concentration of IFN-γ (Figure 6a) and IL-2 (not shown) than T cells from mice uninfected or infected with BCG-261H. To examine the long-term effect of the inoculation of BCG-dHCM on the production of responsive T cells, C57BL/6 mice were subcutaneously inoculated with 1 × 10^3^ CFU/mouse of either BCG-261H or BCG-dHCM 12 weeks before restimulation *in vitro* (Figure 6b). Again, a significantly higher concentration of IFN-γ was produced from splenic T cells obtained from mice inoculated with BCG-dHCM by all stimulators than those from mice uninfected or infected with BCG-261H.

### Effect of BCG-dHCM vaccination on the multiplication of aerosol challenged *M. tuberculosis*

**Figure 7:**
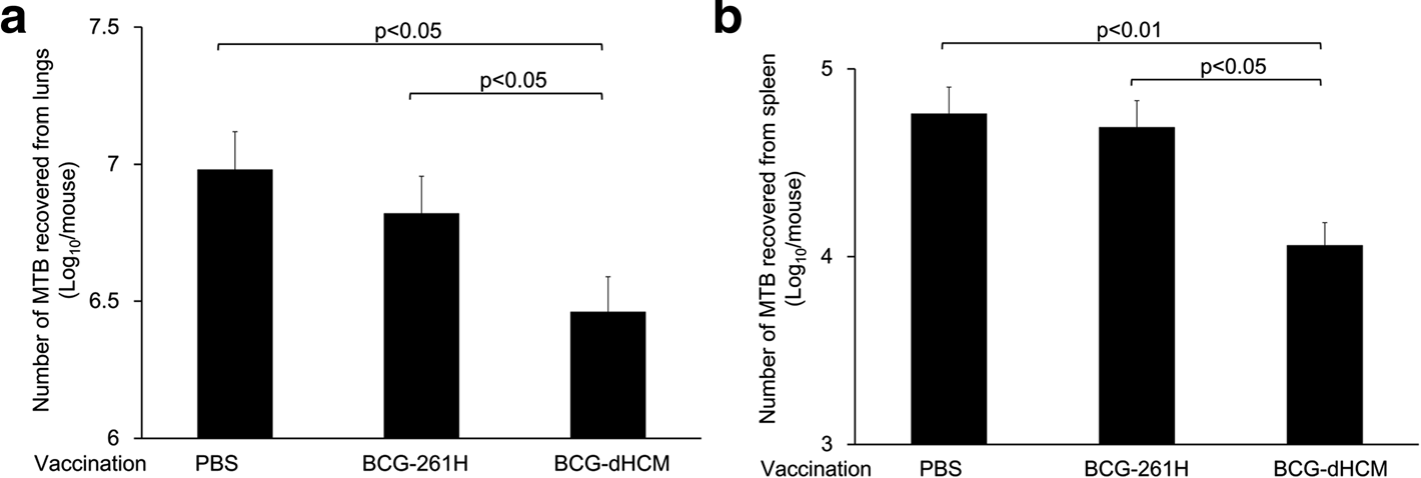
**Inhibition of MTB multiplication by subcutaneous vaccination with BCG-dHCM.** Five-week-old C57BL/6 mice (5 mice/group) were subcutaneously vaccinated with 1 × 10^3^ CFU/mouse of either BCG-261H or BCG-dHCM and were challenged with 100 CFU/lungs of H37Rv by aerosol infection, at 6 weeks post vaccination. The number of MTB recovered from the lung **(a)** or spleen **(b)** at 6 weeks post challenge was enumerated by colony assay method. Representative results of three separate experiments are shown. Titers were statistically compared using Student’s *t*-test.

C57BL/6 mice vaccinated with either BCG-261H or BCG-dHCM (1 × 10^3^ CFU/mouse) for 6 weeks were challenged with 100 CFU per lungs of H37Rv by aerosol infection. Six weeks later, the MTB recovered from both lungs and spleen was enumerated (Figure 7). While the mice vaccinated with BCG-261H minimally inhibit the multiplication of MTB, the vaccination with BCG-dHCM significantly inhibited the growth of H37Rv in lungs (Figure 7a). Similarly, the multiplication of MTB in spleen is significantly inhibited by the vaccination with BCG-dHCM (Figure 7b).

## Discussion

IFN-γ produced by activated type 1 CD4^+^ T cells and CD8^+^ T cells has an essential function in both antimicrobial activity against MTB and limitation of lung inflammation associated with massive accumulation of neutrophils which are recruited by Th17 cells [7-9,46]. Therefore, one of the important aims of vaccination is to produce memory type CD4^+^ T cells as well as memory type CD8^+^ T cells capable of producing abundant dose of IFN-γ by responding rapidly to MTB-infected DC and macrophages. From the studies using T cell receptor-transgenic animal model, it is known that it needs several weeks for host T cells to start inhibiting the multiplication of aerosol infected MTB in primary mice [5,6]. It is unknown whether alveolar APCs could be primed by vaccination, so that time required for APC to move to the regional LN after phagocytosing MTB in lung could be reduced. However, it could certainly reduce the time necessary for T cells to be activated by the interaction with MTB-infected APCs in the LN by differentiating naïve T cells into memory subset by vaccination. BCG has been used widely as a vaccine against tuberculosis, but its effect is quite limited. BCG can only prevent the development of miliary tuberculosis and tuberculosis meningitis in child, but is not effective for prevention of adult lung tuberculosis [15]. However, BCG has many antigenic molecules common to that present in MTB, and moreover, the safety of BCG is well established [16,17]. Thus, we are of the opinion that the improvement of BCG by producing rBCGs would be the fastest route to produce more reliable single injection vaccine against tuberculosis. In this respect, rBCG should have some antigenic molecules which are present in MTB, and also should highly activate not only naïve T cells of both CD4 and CD8 subsets, but also APCs including DC and macrophages. Since both DC and macrophages express MMP-II-related peptide on their surface upon an infection with both H37Ra and H37Rv [31], and also MMP-II can ligate TLR2 and consequently activates NF-κB pathway of APCs [28-31], MMP-II is considered to be good target that could be used as an active vaccinating agent. In this study, we produced new rBCG termed BCG-dHCM, that is a urease-deficient rBCG that secrete the fusion protein composed of HSP70, CysO and MMP-II. In the production of BCG-dHCM, we used MMP-II as a central component, HSP70 as an adjuvant, and CysO as an element necessary to release Ag in APCs. BCG-dHCM secreted the fusion protein in both lysosome in which abundant enzyme is available and in phagosomes of BCG-susceptible cells. Thus BCG-dHCM strongly activated both subsets of naïve T cells and APCs. The efficient activation of APCs by BCG-dHCM is revealed through up-regulation of APC-associated molecules and by enhanced production of various proinflammatory cytokines including IL-12, IL-1β and TNFα from DC and macrophages. The efficient activation of these APCs can be assumed to be directly linked with the effective activation of adaptive immunities. Actually macrophages-infected with BCG-dHCM activated memory type CD4^+^ T cells. Previous rBCG termed BCG-70M that is BCG introduced with HSP70-MMP-II fusion gene failed to activate memory type T cells through macrophages [32], thus, the high immunostimulatory function of BCG-dHCM seems to be owing to the high antigenic load on the surface of BCG-dHCM-infected macrophages, that is due to efficient translocation of BCG-dHCM into lysosome. The effective memory T cells are ones capable of responding to the molecules expressed on the surface of MTB-infected APCs, and are produced by activating naïve T cells in an Ag-specific manner [27,31]. In this respect, the activation of both naïve CD4^+^ T cells and naïve CD8^+^ T cells by BCG-dHCM was dependent on MHC and CD86 molecules expressed on APCs. Thus, BCG-dHCM seems to be stimulating naïve T cells in an Ag-specific fashion. Presumably, APCs infected MTB express various epitopes on their surface, indicating that the presence of clonal diversity of T cells, might provide better control of MTB. Subcutaneous inoculation of BCG-dHCM into C57BL/6 mice produced long lasting T cells responsive to *in vitro* secondary stimulation. Upon stimulation with not only MMP-II, CysO and HSP70, but also H37Rv-derived cytosolic (Figure 6) and membrane (not shown) protein, T cells obtained from mice inoculated with BCG-dHCM were efficiently reactivated and produced high concentration of IFN-γ. Although the exact reason why these T cells were capable of responding to MTB components is not clear, it may be reasonable to speculate that the translocation of BCG-dHCM into lysosome induced the degradation of BCG that leads to production of epitope derived from BCG itself. CysO is engaged in alternative cysteine biosynthesis pathway, which plays an essential role for the MTB survival in the oxidative condition [33,34]. Also, CysO is categorized in ubiquitin superfamily and is possible to direct protein towards proteosome degradation pathway [37]. In fact, in this study, we found that rCysO, produced by using *M. smegmatis* in a LPS-free condition, activated both innate and adaptive cellular responses, since it induced some cytokine production from APCs and phenotypic changes in DC, and also stimulated IFN-γ production from both subsets of T cells. When we introduced *CysO* gene into urease-deficient rBCG accompanying with HSP70-MMP-II fusion gene, CysO was produced as a part of fusion protein, in fact, the immunogenic function of CysO may be up-regulated because of the chaperone activity of HSP70, that may lead to production of CysO-specific memory T cells *in vivo*, although we could not elucidate the detailed mechanisms of the immunological function of CysO*.* Actually, mice inoculated with BCG-dHCM produced T cells that respond to CysO vigorously. These results indicate that CysO protein was certainly secreted from rBCG in APC and was used for T cell activation. CysO expression in MTB is inhibited in hypoxic condition and is induced by reaeration [34], thus, CysO may be highly expressed in MTB in active phase, and, also, is involved in the survival of MTB in macrophages. Therefore, production of CysO reactive T cells may be advantageous for the induction of host defense reaction against MTB which is re-activated in macrophages. In this study, we used small dose of BCG, one fifth of usual dose [47], and by which the parent BCG cannot inhibit MTB growth in lungs in our hands (not shown), for vaccination of mice in order to elucidate the difference between BCG-261H and BCG-dHCM. As expected BCG-261H did not inhibit the MTB growth, but even a small dose of BCG-dHCM at least partially inhibits multiplication of MTB in both lungs and spleen. In this respect, BCG-dHCM seems to be superior to BCG-261H. The reason why BCG-261H did not inhibit the multiplication of MTB may be because we tested bacterial burden in lung and spleen at 6 wks, but not 4 wks which is a frequently used time point [47], after MTB challenge, because report suggests that 4 ~ 5 wks are necessary to reach the stable level of pulmonary bacterial burden even in naïve mice [6]. Also, due to BCG strain differences, there may be differences in protective effect in mice experiments. However, more detailed studies, for example, the difference between BCG-DHTM and BCG-dHCM, and the relation between the presence of CysO-reactive T cells and MTB replication in macrophages, are absolutely required, to prove the usefulness of CysO involvement for the production of vaccine against tuberculosis.

## Conclusions

These results indicate that the secretion of polycomponent antigenic molecules can efficiently produce polyclonal Ag-specific T cells responsive to secondary stimulation *in vivo*, and may provide one possible tool for the development of better vaccine against tuberculosis.

## Abbreviations

BCG: *Mycobacterium bovis* BCG; M: *Mycobacterium*; r: Recombinant; MTB: *M. tuberculosis*; HSP: Heat shock protein; MMP: Major membrane protein; IFN-γ: Interferon-gamma; DC: Dendritic cells; Ag: Antigen; MHC: Major histcompatibility complex; APC: Antigen-presenting cell; TLR: Toll-like receptor; PBMC: Peripheral blood mononuclear cell; mAb: Monoclonal antibody; MOI: Multiplicity of infection; CFU: Colony forming unit.

## Competing interests

The authors declare that they have no competing interests.

## Authors’ contribution

YT and MM participated in the design of the study and carried out the cell culture experiments, YM and TT carried out animal studies, YT and TM conducted the construction of recombinant BCG. YT, YM and MM were involved in the preparation of the manuscript. All authors have read and approved the final manuscript.

## Pre-publication history

The pre-publication history for this paper can be accessed here:

http://www.biomedcentral.com/1471-2334/14/179/prepub
